# DMXL2 Is Required for Endocytosis and Recycling of Synaptic Vesicles in Auditory Hair Cells

**DOI:** 10.1523/JNEUROSCI.1405-23.2024

**Published:** 2024-08-15

**Authors:** Hu Peng, Longhao Wang, Yunge Gao, Huihui Liu, Guotong Lin, Yu Kong, Pengcheng Xu, Hongchao Liu, Qingyue Yuan, Huanhai Liu, Lei Song, Tao Yang, Hao Wu

**Affiliations:** ^1^Department of Otolaryngology–Head and Neck Surgery, Shanghai Ninth People’s Hospital, Shanghai Jiaotong University School of Medicine, Shanghai 200023, China; ^2^Ear Institute, Shanghai Jiaotong University School of Medicine, Shanghai 200125, China; ^3^Shanghai Key Laboratory of Translational Medicine on Ear and Nose Diseases, Shanghai 200125, China; ^4^Department of Otolaryngology–Head and Neck Surgery, Changzheng Hospital, Naval Medical University, Shanghai 200003, China; ^5^Institute of Neuroscience, CAS Center for Excellence in Brain Science and Intelligence Technology, Chinese Academy of Sciences, Shanghai 200031, China

**Keywords:** DFNA71, DMXL2, endocytosis, hair cell, hearing loss, synaptic vesicle

## Abstract

Ribbon synapses of inner hair cells (IHCs) are uniquely designed for ultrafast and indefatigable neurotransmission of the sound. The molecular machinery ensuring the efficient, compensatory recycling of the synaptic vesicles (SVs), however, remains elusive. This study showed that hair cell knock-out of murine *Dmxl2*, whose human homolog is responsible for nonsyndromic sensorineural hearing loss DFNA71, resulted in auditory synaptopathy by impairing synaptic endocytosis and recycling. The mutant mice in the C57BL/6J background of either sex had mild hearing loss with severely diminished wave I amplitude of the auditory brainstem response. Membrane capacitance measurements of the IHCs revealed deficiency in sustained synaptic exocytosis and endocytic membrane retrieval. Consistent with the electrophysiological findings, 3D electron microscopy reconstruction showed reduced reserve pool of SVs and endocytic compartments, while the membrane-proximal and ribbon-associated vesicles remain intact. Our results propose an important role of DMXL2 in hair cell endocytosis and recycling of the SVs.

## Significance Statement

The molecular basis underlying efficient recycling of the ribbon synaptic vesicles (SVs) in cochlear hair cells remains elusive. In this study, investigation of a hair cell-specific knock-out mouse for Dmxl2 identifies its import roles in endocytosis and recycling of the auditory SVs. The mutant mice display auditory synaptopathy, a common feature for noise-induced hearing loss and age-related hearing loss. The inner hair cells show deficiency in sustained synaptic exocytosis and endocytic membrane retrieval with reduced reserve pool of SVs and endocytic compartments. Our results provide new insights into the molecular machinery ensuring ultrafast and indefatigable neurotransmission of the sound.

## Introduction

Sound signals are encoded by ribbon synapses in the cochlear inner hair cells (IHCs) with remarkable temporal precision at the submillisecond level (Palmer and Russell, 1986). Compared with most conventional synapses, IHC ribbon synapse can operate at much higher speed by about two orders of magnitude (Moser and Beutner, 2000; Fernandez-Alfonso and Ryan, 2004). The fast release of the glutamate neurotransmitter by IHC synaptic vesicles (SVs) is sustainable as long as the sound continues, requiring both rapid exocytosis and efficient resupply of the readily releasable pool (RRP) of SVs at the active zone (Griesinger et al., 2005).

To accommodate the need for ultrafast, indefatigable neurotransmitter release during sound processing, IHC ribbon synapses adopt a specialized molecular machinery distinct from that of the conventional synapses. Many essential exocytic proteins for other neuronal cells, such as synaptophysin, synapsins, complexins, soluble *N*-ethylmaleimide-sensitive factor attachment protein receptor proteins, and Munc13 and CAPS proteins, are not expressed in IHCs (Safieddine and Wenthold, 1999; Strenzke et al., 2009; Nouvian et al., 2011; Vogl et al., 2015). Instead, the multi-C2 domain protein otoferlin, whose encoding gene *OTOF* is responsible for human recessive deafness DFNB9, expresses in IHCs in close correlation with afferent synaptogenesis and plays a central role in Ca^2+^-triggered synaptic exocytosis (Roux et al., 2006). At IHC ribbon synapses, neurotransmission is driven by Ca^2+^ influx through Ca_V_1.3 channels, which have characteristically little Ca^2+^-dependent inactivation (Cui et al., 2007). Otoferlin has been shown to interact with Ca_V_1.3 (Hams et al., 2017) and act as the major Ca^2+^ sensor for SV fusion and prefusion priming (Roux et al., 2006; Pangrsic et al., 2010), enabling fast release and replenishment of RRP. In addition, otoferlin is also involved in postfusion release site clearance by interaction with the clathrin adapter protein AP-2μ (Jung et al., 2015), and in reformation of SV from the endosome-like vacuoles (ELVs), likely via endocytic bulk membrane retrieval (Strenzke et al., 2016).

To sustain the high rates of neurotransmission in IHCs, exocytosis must be followed by coordinated, equally efficient endocytosis and recycling of the SVs, whose molecular regulation remains poorly understood. Genetic disruption of conventional endocytic proteins synaptojanin 1, dynamin 1, AP-2μ, or endophilin-A in mouse or zebrafish hair cells resulted in impaired endocytic membrane retrieval (dynamin 1 and endophilins), stalled SV reformation following bulk endocytosis (AP-2μ and endophilin-A), or reduced reserve pool of vesicles (synaptojanin 1), suggesting their involvement in synaptic endocytosis and recycling in IHCs (Trapani et al., 2009; Neef et al., 2014; Jung et al., 2015; Kroll et al., 2019). Though auditory synaptopathy has been increasingly recognized in genetic and acquired sensorineural hearing loss (Moser and Starr, 2016), few deafness-related genes identified in humans have been functionally implicated in SV endocytosis and recycling.

Previous human genetic studies identified mutations in *DMXL2* as the pathogenic causes for dominant nonsyndromic hearing loss DFNA71 and recessive syndromic hearing loss associated with polyendocrine-polyneuropathy syndrome and developmental and epileptic encephalopathy named Ohtahara syndrome (Tata et al., 2014; Chen et al., 2017; Esposito et al., 2019; Wonkam-Tingang et al., 2021). *DMXL2* encodes the α subunit of rabconnectin 3 whose expression is strongly associated with SVs in the brain. The α and β subunits of rabconnectin 3, encoded by *DMXL2* and *WDR7*, respectively, form a protein complex with Rab3 GEP and Rab3 GAP (Nagano et al., 2002; Kawabe et al., 2003), regulators of Rab3 GTPases that are involved in docking and priming of SVs during endocytosis (Hutagalung and Novick, 2011). Loss of DMXL2 results in neurodevelopmental defects associated with impaired neuronal autophagy and synaptic connection (Esposito et al., 2019). In addition, DMXL2 regulates the assembly and activity of the v-ATPase proton pumps that acidifies various intracellular organelles, including SVs and endosome, for their proper function in a variety of eukaryotic cells (Yan et al., 2009; Einhorn et al., 2012; Tuttle et al., 2014). *Dmxl2* mutant zebrafish has reduced auditory escape behavioral response and defective hair cell SV acidification and synaptic transmission, though the impact of *Dmxl2* deficiency on synaptic membrane trafficking remains to be resolved (Einhorn et al., 2012). Our previous study detected enriched expression of DMXL2 in the basolateral region of IHCs in the mouse cochlea where intensive SV trafficking and fusion occurs (Chen et al., 2017). In the current study, investigation of a hair cell-specific conditional knock-out (CKO) mouse model showed that *Dmxl2* plays import roles in SV endocytosis and recycling in auditory hair cells.

## Materials and Methods

### Generation of the *Dmxl2* hair cell-specific CKO mice

The *Dmxl2^fl/fl^* mice were generated in the C57BL/6J background using the CRISPR–Cas9 system. Briefly, a mixture of active guide RNA molecules, two single-stranded oligo donor nucleotide donors, and Cas9 protein was injected into the cytoplasm of C57BL/6J embryo. Two loxP sequences were inserted into introns 1 and 4 of *Dmxl2* through homology-directed repair after Cas9 creates double-stranded breaks at the designated sites. Constitutive, hair cell-specific, and outer hair cell (OHC)-specific knock-outs of *Dmxl2* were achieved by crossing the *Dmxl2^fl/fl^* mice with the *Actb*^cre/+^, *Gfi1*^cre/+^, and *Slc26a5*^cre/+^ mice, respectively. Both male and female mice were studied. All experiments were performed following the national animal care guidelines and approved by the animal welfare committee of the Ninth People's Hospital, Shanghai Jiaotong University School of Medicine.

### Balanced beam tests

Balanced beams of 10 and 20 mm in width, 1 m in length, and 2 mm in height were placed between two dark boxes. The beginning and ending points for measurement were set as 75 cm apart on the beams. Before the formal tests, the mice were trained on beams for 3 consecutive days, five times per day. The formal tests measured the time on the beam across the beginning and ending points.

### Rotarod tests

Mice were placed on a rod of 30 mm in diameter and 60 mm in length in enclosed housing. Before the formal tests, the mice were trained on rods for 4 consecutive days at increasing rotating rates of 10, 20, 30, and 40 rpm. The formal tests were conducted at the fifth day at 40 rpm. Each test consisted of three consecutive runs of 5 min with recovering time of at least 1 h in between. The length of the time the mice remain on the rod before falling was recorded.

### Auditory brainstem responses

For auditory brainstem response (ABR) recording, mice were anesthetized with 480 mg/kg ketamine i.p. The core temperature was maintained at 37°C during the recording process using the Homeothermic Monitoring System (Harvard Apparatus). Hearing thresholds at sound frequencies 4, 5.6, 8, 11, 16, 22, 32, and 45 kHz were assessed using the TDT III system (Tucker-Davis Technologies) in a soundproof chamber. The active, reference, and ground electrodes were subcutaneously positioned at the vertex, left mastoid, and right shoulder, respectively. Pure tone bursts were produced in the free field using a MF1 speaker 10 cm from the vertex. The evoked potentials were filtered with a bandpass filter from 32 to 4,000 Hz and averaged 400 times. For each tested frequency, the sound level was reduced from 90 to 0 dB SPL in 5 dB steps. ABR thresholds were determined by minimal stimulus level that evoked a visible and reproducible response waveform. Amplitudes and latencies of the ABR wave I were measured and analyzed using the BioSigRZ software (Tucker-Davis Technologies).

### Distortion product otoacoustic emission

The distortion product otoacoustic emission (DPOAE) tests were performed using a small ER10B + microphone (Etymotic Research) attached to the right external auditory meatus of the mice. Primary tones (f1 and f2, with f2 / f1 = 1.2) of equal intensity were generated by two EC1 electrostatic speakers (Tucker-Davis Technologies). Distortion product data at the frequency 2f1–f2 was measured every 21 ms and averaged 512 times in response to center frequencies at 5.6, 8, 11.2, 16, 22.4, and 32 kHz with 5 dB increments from 20 to 80 dB SPL. The DPOAE thresholds were defined as the point where the distortion product can no longer be distinguished from the noise of the microphone.

### Whole-cell patch-clamp recordings

The apical turn of the basilar membrane of the mouse cochlea was microdissected in the extracellular solution. Whole-cell patch-clamp recordings were performed using the EPC10/2 amplifier (HEKA Electronics) with the Patchmaster software (HEKA Electronics) as described in our previous studies (Lin et al., 2019; Liu et al., 2019a; Zhao et al., 2020). Current–voltage relationships of Ca^2+^ influx in IHCs were obtained from current responses to ramp depolarization from −90 to 60 mV and fitted to the following equation:

$$I(V)=(V-V_{\mathrm{rev}})\times \frac{G_{\max}}{1+\exp(-(V-V_{\mathrm{half}})/K_{\mathrm{slope}})},$$

where *V* is the command membrane potential, *V*_rev_ is the reversal potential, *G*_max_ is the maximum conductance, *V*_half_ is the half activation potential, and *K*_slope_ is the steepness of voltage dependence in current activation. IHC capacitance measurement (*C*_m_) was performed with the lock-in feature and the “Sine + DC” method in the software Patchmaster (Liu et al., 2019b). Briefly, a 1 kHz sine wave and a 70 mV peak-to-peak magnitude were superposed on the IHC holding potential of −90 mV. The averaged capacitances change before and after the depolarization was calculated to monitor exocytosis from IHCs: △*C*_m _= *C*_m_ (response) − *C*_m_ (baseline). Ca^2+^ current charge (*Q*_ca_) was calculated by taking the integral of the leak-subtracted current during depolarization.

### Nonlinear capacitance

We recorded whole-cell patch-clamped OHCs for nonlinear capacitance (NLC), a surrogate measurement of electromotility. The membrane-holding potential of the NLC measurement was set at 0 mV. A 10 mV two-sine stimulus (390.6 and 781.2 Hz) was superimposed onto a 300 ms voltage ramp from +160 to −160 mV. The first derivative of a two-state Boltzmann function was fitted to the following equation:

$$C_{m}=Q_{\max}\frac{ze}{\mathrm{kT}}\;\frac{b}{{\left(1+b\right)}^{2}}\;\;+C_{\mathrm{lin}},$$

where

$$b\;=\;\exp\left(\frac{-ze(V_{m}-V_{\mathrm{pk}C_{m}})}{kT}\right),$$

where *Q*_max_ is the maximum nonlinear charge moved, *V*_pkCm_ is the voltage at peak capacitance or equivalent to half maximum charge transfer, *V*_m_ is the membrane potential, *z* is the valence, *C*_lin_ is the linear membrane capacitance, *e* is the electron charge, *k* is Boltzmann’s constant, and *T* is the absolute temperature.

### FISH, RNAscope, and immunofluorescence staining of the mouse organs of Corti

For FISH, the organ of Corti was dissected and fixed in 4% paraformaldehyde (DEPC) for 1 h, digested with proteinase K (20 μg/ml) at 37°C for 5 min, prehybridized with the hybridization buffer at 37°C for 1 h, and hybridize in a humidity chamber overnight.

All reagents and instruments need to be RNase free. Probes are targeted to or outside of the deleted exons 2–4 region of *Dmxl2* (see probe sequences in Extended Data Table 1-1).

For RNAscope, apply 2–4 drops per slide of RNAscope® hydrogen peroxide for 10 min at room temperature, then add 2–4 drops of RNAscope Protease III, and incubate for 30 min. Perform RNAscope® multiplex fluorescent v2 assay (No. 323100, ACD) following the manufactory manual combined with the Immunofluorescence – Integrated Co-Detection Workflow. RNAscope® probes used in this study include Mm-*Otof* C2 (No. 485671, ACD), Mm-*Sh3gl2* (No. 492641, ACD), Mm-*Synj1* (No. 829951, ACD), Mm-*Dnm1* (No. 446931, ACD), and Mm-*Rab3a* (No. 518171, ACD).

For immunofluorescence staining, the organs of Corti were fixed with 4% paraformaldehyde at 4°C overnight. Whole-mount samples were permeabilized with 1% Triton X-100 for 1 h and blocked with 5% bovine serum albumin for 2 h at room temperature. Primary antibodies were applied at 4°C overnight, which included mouse anti-CTBP2 (BD-612044, BD Biosciences), rabbit anti-GLUR2 (SAB4501295, Merck Millipore), rabbit anti-DMXL2 (PA5-100477, Invitrogen), rabbit anti-WDR7 (NBP2-14517, Novus Biologicals), rabbit anti- MYO7A antibody (25-6790, Proteus BioSciences),.and rabbit anti-RAB3A (ab3335, Abcam). Secondary Alexa Fluor-labeled antibodies (Invitrogen) were applied at room temperature for 2 h. Images were captured by the Zeiss LSM 880 laser confocal microscope (Carl Zeiss Microscopy). For quantitative immunostaining of the ribbon synapse, *Z*-stack imaging was achieved using the ZEN software (Carl Zeiss Microscopy).

### Transmission electron microscopy

Cochleae were quickly dissected and fixed immediately in 2% glutaraldehyde at 4°C overnight. On ice, the basilar membrane samples were postfixed with 1% osmium tetroxide for 1 h, stained with 1% uranyl acetate for 1 h, and dehydrated through increasing ethanol concentrations. After being embedded in AGAR-100, ultrathin sections of 70–75 nm were generated with a Diatome 35° diamond knife using the Ultracut E ultramicrotome (Leica Microsystems). The ultrathin sections were then placed on the 1% formvar-coated copper mesh grids and poststained with uranyl acetate and lead citrate. The samples were imaged using a JEM-1011 transmission electron microscope (JEOL) with a Gatan Orius 1200A camera (Gatan) at 10,000-fold magnification.

### Automated tape-collecting ultramicrotome scanning electron microscopy imaging

After fixation, ultrathin sections of 40 nm were continuously cut and placed on the silicon chip in order, which cover at least three consecutive IHCs. Images of the whole-cell scale were first obtained and analyzed with the 10 nm resolution. Regions of interest were then selected, scanned, and presented with the 2 nm resolution. The final image resolution for ribbon and vesicle analysis is 2 nm. The image analysis was performed using the Dragonfly software (Object Research Systems). The number and size of the SVs were calculated as previously described (Jean et al., 2018).

### Date processing and statistical analysis

Imaging data processing and statistical analyses were carried out with Igor Pro (WaveMetrics) and GraphPad Prism 8.0 (GraphPad software). Comparisons between two experiment groups were performed using the two-tailed, unpaired, or paired Student's *t* test. For comparisons of multiple experiment groups, one-way or two-way ANOVA was used followed by Bonferroni’s post hoc test. Comparisons of cumulative distribution were performed using the Kolmogorov–Smirnov test. Data were shown as mean ± SDV unless stated otherwise. Differences were considered statistically significant when *p *< 0.05. In figures, n.s. represents *p *> 0.05, ∗ represents *p *< 0.05, ∗∗ represents *p *< 0.01, ∗∗∗ represents *p *< 0.001, and *n* represents the number of animals, cells, or recordings.

## Results

### The *Dmxl2* CKO mice have mild hearing loss with severely diminished wave I amplitude of the auditory brainstem responses

A floxed *Dmxl2^fl/fl^* mouse line was generated by inserting two loxP sites in the mouse genome flanking exons 2–4 of *Dmxl2*. Constitutive knock-out of *Dmxl2* (*Dmxl2^fl/fl^*; *Actb*^cre/+^) is embryonically lethal. To study the presynaptic function of DMXL2 in mammalian auditory hair cells, a previously described *Gfi1*^cre/+^ mouse line was crossed to knock-out *Dmxl2* specifically in the hair cells (Yang et al., 2010). Because the *Gfi1*^cre/+^ mouse has inherent early onset, progressive hearing loss (Matern et al., 2017), we use the *Dmxl2^/+/+^*; *Gfi1*^cre/+^ mouse as controls in all subsequent experiments.

**Figure 1.**
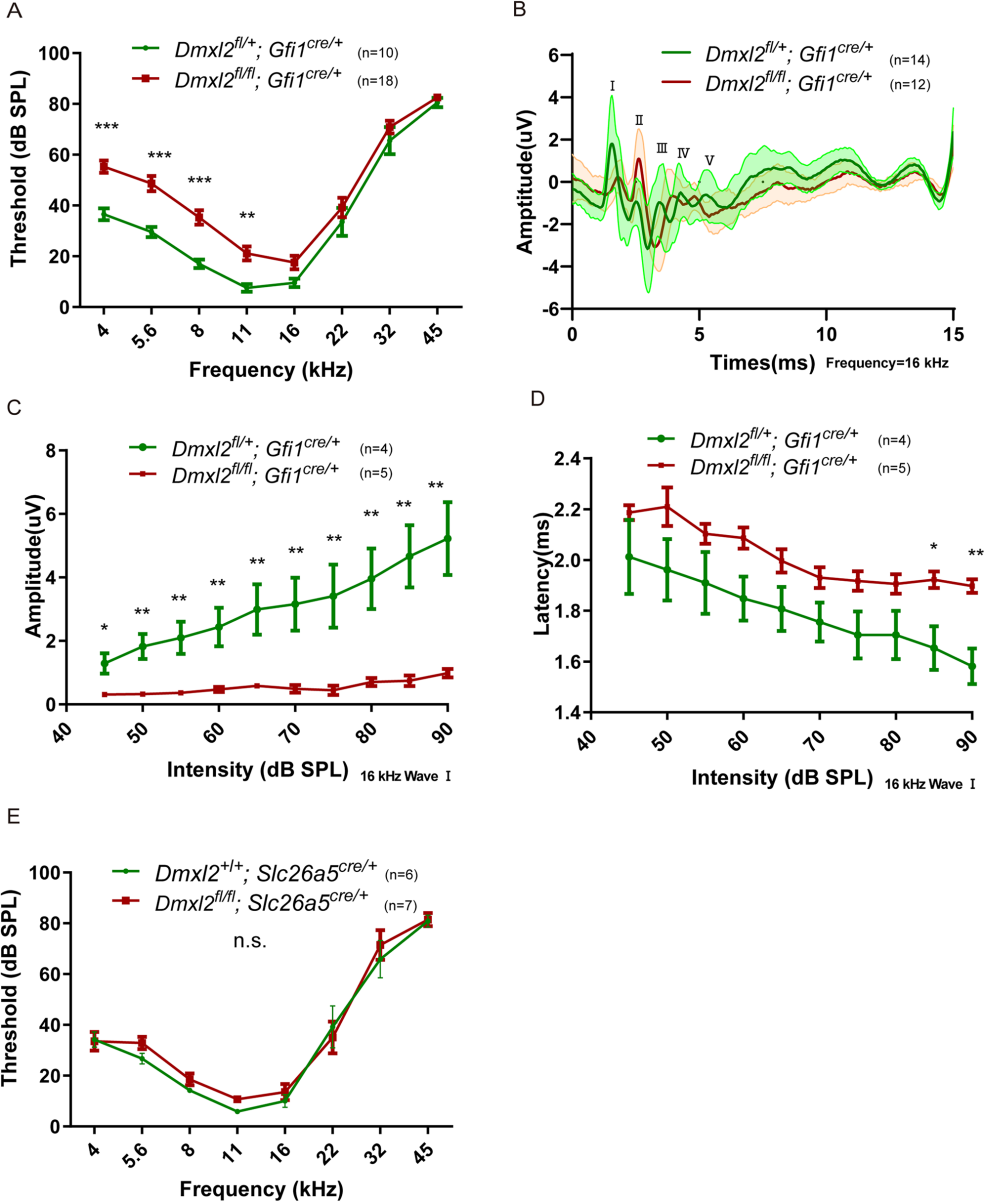
ABR of the *Dmxl2* CKO and control mice at 1 month of age. ***A***, The *Dmxl2* CKO mice have elevated ABR thresholds of 20 dB or less between 4 and 11 kHz. ***B–D***, Suprathreshold wave I amplitudes are severely decreased in the CKO mice at representative frequencies of 16 kHz, while the wave I latencies are longer when tone stimulus intensity is 85 dB SPL or higher. ***E***, ABR thresholds were unaltered in the *Dmxl2^fl/fl^*; *Slc26a5*^cre/+^ mice. *n*: animal numbers. Data are presented as the mean ± SEM and statistically analyzed using two-way ANOVA followed by Bonferroni’s post-test. The *Dmxl2* CKO and control mice are also evaluated for their vestibular function by the balanced beam tests and the rotarod tests (Extended Data Fig. 1-1) and their OHC function by the DPOAE and the NLC analysis (Extended Data Fig. 1-2) at 1 month of age.

Both balanced beam and rotarod tests showed that the *Dmxl2* CKO mice have normal vestibular function (Extended Data Fig. 1-1, *n* = 6 each, *p *> 0.05). Recordings of ABR showed that the *Dmxl2* CKO mice have mild hearing loss at 1 month of age, with elevation of ABR thresholds equal or <20 dB between 4 and 11 kHz (Fig. 1*A*, *n* = 10–18 each, *p *< 0.001 for frequencies 4, 5.6, and 8 kHz, *p *< 0.01 for frequency 11 kHz). On the other hand, wave I of the ABR has severely diminished amplitude, while amplitudes of waves II to V are better preserved (Fig. 1*B*, *n* = 12–14 each, and Fig. 1*C*, *n* = 4–5 each, *p *< 0.05 for intensity 45 dB SPL, *p *< 0.01 for intensity 50–90 dB SPL), a feature reflecting reduced compound action potential of the spiral ganglion neurons. The hearing loss and reduced ABR amplitudes were not seen in the *Dmxl2^fl/fl^*; *Slc26a5*^cre/+^ mice with targeted knock-out of *Dmxl2* in the OHCs only (Fig. 1*E*, *n* = 6–7 each, *p *> 0.05), and the DPOAE and the NLC of the *Dmxl2* CKO mice were normal (Extended Data Fig. 1-2*A*, *n* = 4 each, *p *> 0.05, and Extended Data Fig. 1-2*C–F*, *n* = 9–12 each, *p *> 0.05), suggesting that the lesion primarily comes from the IHCs instead of the OHCs.

10.1523/JNEUROSCI.1405-23.2024.f1-1 Figure 1-1 Vestibular functions of the *Dmxl2* CKO and control mice at one month of age. (A) Time to cross the beam is indistinguishable between the *Dmxl2* CKO and control mice in the balanced beam tests. (B) Time to fall is indistinguishable between the *Dmxl2* CKO and control mice in the rotarod tests. n: animal numbers. Groups are compared using paired *t*-test. Download Figure 1-1, TIF file.

**Figure 2.**
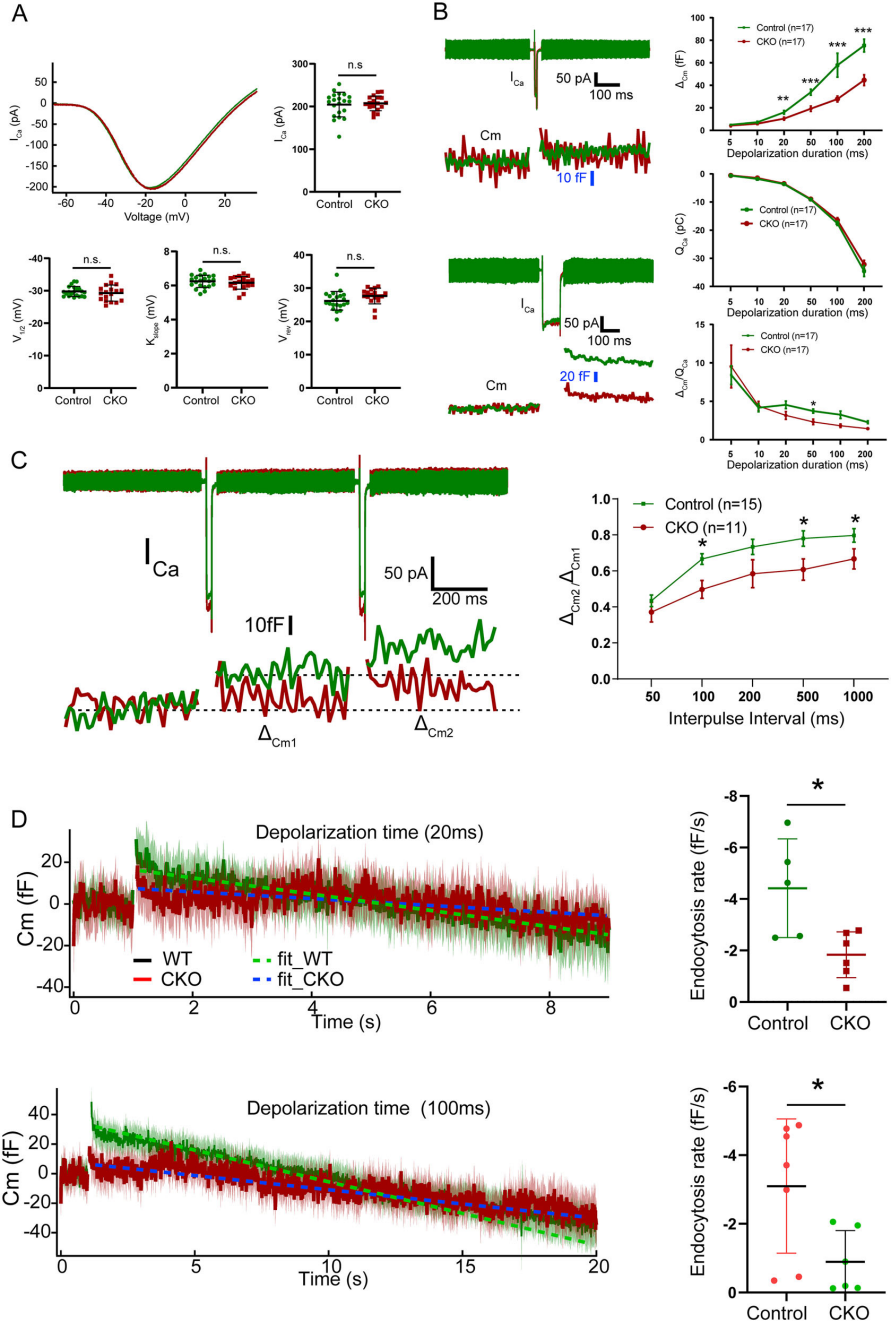
Patch-clamp recordings of IHCs in the *Dmxl2* CKO and control mice at 1 month of age. ***A***, Ca^2+^ influx of the CKO and control IHCs has comparable current–voltage curve, current amplitude (*I*_Ca_), and voltage dependence (assessed based on *V*_1/2_, *K*_slop_, and *V*_rev_). ***B***, Left panels show representative Ca^2+^ currents and corresponding membrane capacitance (*C*_m_) traces recorded in response to 20 ms (top) and 100 ms (bottom) depolarization. Right panels show Δ*C*_m_ (top), Q_ca_ (middle), and Δ*C*_m_/*Q*_Ca_ (bottom) evoked by depolarizing pulses with durations ranging from 5 to 200 ms. Assessed based on Δ*C*_m_ and the Δ*C*_m_/*Q*_Ca_ ratio, exocytosis under prolonged stimulation is significantly reduced in IHCs of the CKO mice. *n*: IHC numbers; the animal numbers for WT and CKO groups are 5 and 6, respectively. ***C***, Representative traces (left) collected from the double-pulse stimulation protocol show differential Δ*C*_m_ evoked by two consecutive 20 ms depolarizations. The ratio of Δ*C*_m2_/Δ*C*_m1_ (right) is reduced in the CKO IHCs, indicating decreased efficiency for SV replenishment. *n*: IHC numbers; the animal numbers for WT and CKO groups are 8 and 6, respectively. ***D***, Endocytosis is assessed by determining the decrease in *C*_m_ after 20 ms (top) and 100 ms (bottom) depolarizing pulses and fitting with a linear function. The CKO IHCs showed a slower linear *C*_m_ recovery after 20 and 100  ms depolarization. The animal numbers for WT and CKO groups are 4 and 5, respectively. The data are presented as the mean ± SEM and analyzed using unpaired *t* test (***A***, ***D***) and two-way ANOVA followed by Bonferroni’s post-test (***B***, ***C***).

10.1523/JNEUROSCI.1405-23.2024.f1-2 Figure 1-2 OHC functions of the *Dmxl2* CKO and control mice at one month of age. (A) The *Dmxl2* CKO mice have normal DPOAE thresholds. n: animal numbers. (B-F) Whole cell patch clamped OHCs of the *Dmxl2* CKO mice show normal NLC trace, Q_max_, C_lin_, and normalized prestin’s charge density Q_sp_ derived from Qmax/Clin, suggesting normal OHC electromotility. n: OHC numbers; the animal numbers for WT and CKO groups are 5 each. Data are presented as the mean ± SEM and statistically analyzed using unpaired *t*-test. Download Figure 1-2, TIF file.

### DMXL2 deficiency impairs SV replenishment and endocytic membrane retrieval in IHCs

We next performed perforated patch-clamp recordings to examine the presynaptic function of DMXL2 in IHCs. At 1 month of age, the CKO IHCs have unaltered current–voltage relationship (Fig. 2*A*, *n* = 21 each, *p *> 0.05), which reflects normal Ca_V_1.3-mediated Ca^2+^ influx. Exocytic membrane capacitance increments (Δ*C*_m_) are normal in response to short depolarization of 5–10 ms (Fig. 2*B*, *n* = 17 each, *p *> 0.05 for 5 and 10 ms), suggesting intact fusion of the RRP. Sustained exocytosis for depolarization of 20–200 ms, however, is significantly reduced (Fig. 2*B*, *n* = 17 each, *p < *0.01 for 20 ms, *p < *0.001 for 50, 100, and 200 ms), indicating impaired replenishment of the SVs to RRP. RRP recovery from depletion, assessed as the paired-pulse ratio (Δ*C*_m2_/Δ*C*_m1_) for various interpulse intervals, is also deficit in the CKO IHCs (Fig. 2*C*, *n* = 11–15 each, *p < *0.01 for 100, 500, and 1,000 ms). Importantly, *C*_m_ declines after exocytosis, induced by both short (20 ms) and long (100 ms) depolarization, is slower in CKO IHCs (Fig. 2*D*, *n* = 5–7 each, *p < *0.05), indicating compromised endocytic membrane retrieval through clathrin-mediated endocytosis and possibly bulk retrieval (Neef et al., 2014). The combined electrophysiological results suggest an important role of DMXL2 in SV replenishment and recycling in IHCs.

### DMXL2 deficiency results in reduced reserve pool of SVs and endocytic compartments in IHCs

**Figure 3.**
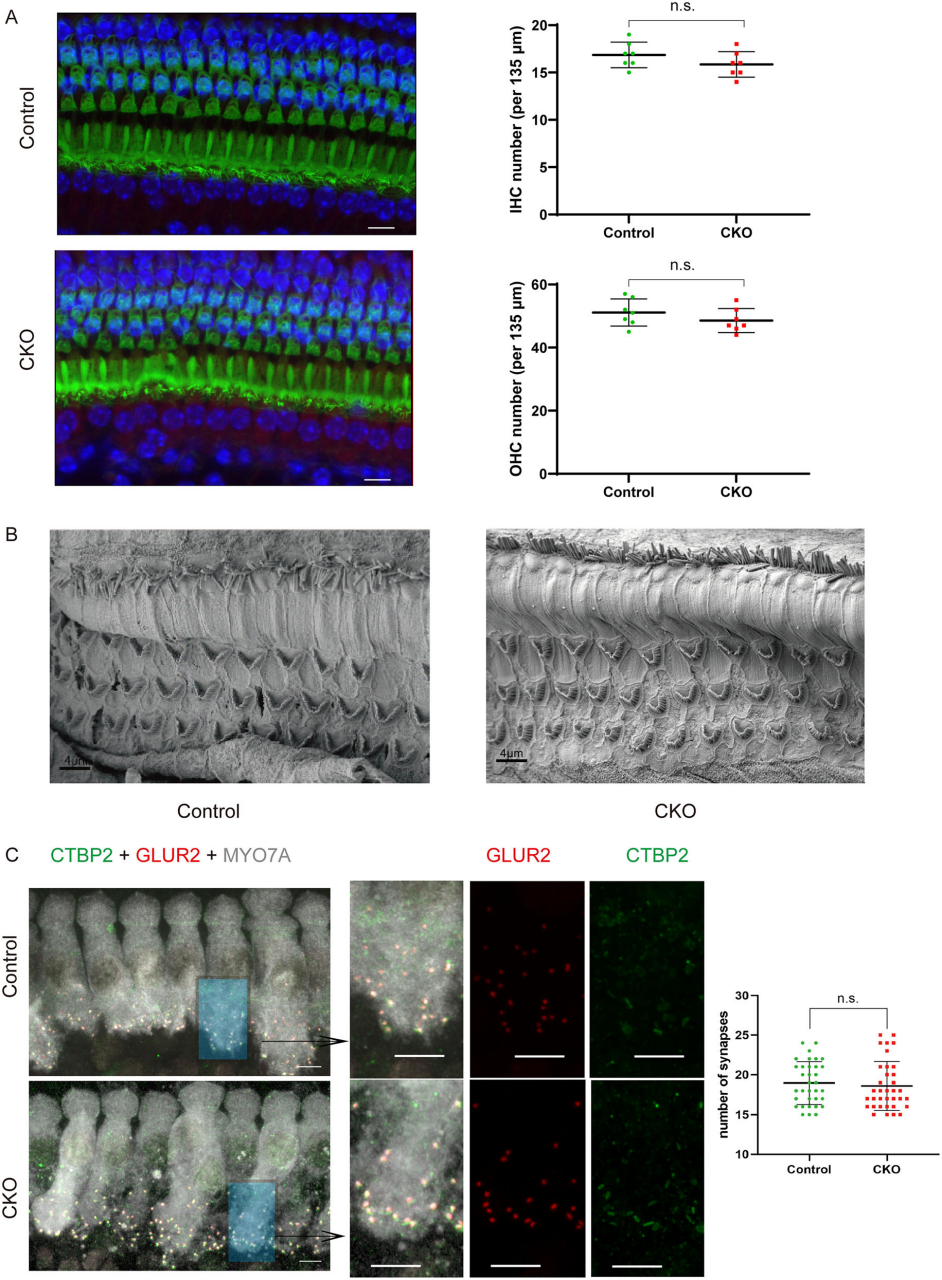
Hair cell morphology and subcellular features of the *Dmxl2* CKO and control mice at 1 month of age. ***A***, Immunofluorescence staining showing the normal hair cell counts of the CKO mice in the middle turn of the cochlea. Scale bars: 10 μm. ***B***, Scanning electron microscopy showing the normal stereocilia morphology of the CKO hair cells. ***C***, Immunofluorescence staining showing the normal ribbon synapse count of the CKO IHCs by double staining of anti-CTBP2 (green) and anti-GLUR2 (red), the presynaptic and postsynaptic markers for ribbon synapses. Gray: anti-MYO7A. Scale bars: left, 5 μm; right, 4 μm. Examined in three mice each in WT and CKO groups. The data are statistically analyzed using unpaired *t* test.

**Figure 4.**
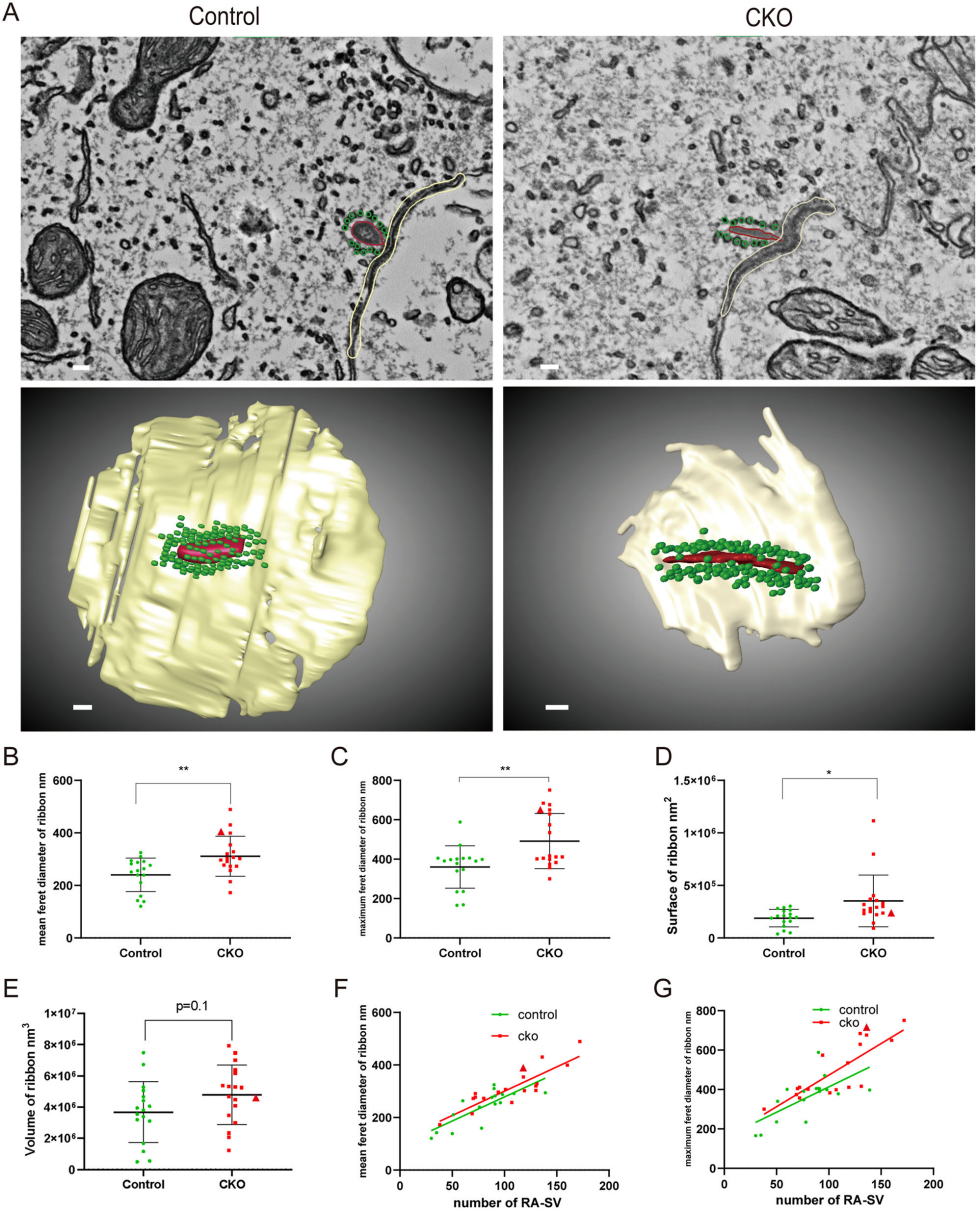
IHC ribbon morphology and RA-SV counts of one representative *Dmxl2* CKO mouse and control mouse (17 ribbons each) at 1 month of age by ATUM-SEM. A subgroup of the *Dmxl2* CKO ribbons is elongated and has proportionally increased number of RA-SVs. ***A***, Sectional images (top) and reconstructed models (bottom) based on the ATUM-SEM data showing representative normal control ribbon and elongated CKO ribbon (red) and their RA-SVs (green). Light brown: surface of the cytoplasmic membrane with the ribbons in proximity. Scale bars: 100 nm. ***B–E***, Comparison of the length, volume, and surface of the ribbons shows that a subgroup of CKO ribbons is elongated in comparison with the control ribbons. Feret’s diameter, defined as the distance between two parallel tangents of the particle at angles separated by 10° from each other (Slobodan et al., 2016), was used for length measurement of the irregular-shaped ribbons. The elongated CKO ribbon shown in panel ***A*** is marked as a triangle in panels ***B–E***. ***F***, ***G***, The number of RA-SVs is in proportion to the mean and maximum Feret’s diameters of the ribbons and slightly increased in IHCs of the CKO mice. Examined in one representative control and CKO mice. The data are statistically analyzed using unpaired *t* test.

**Figure 5.**
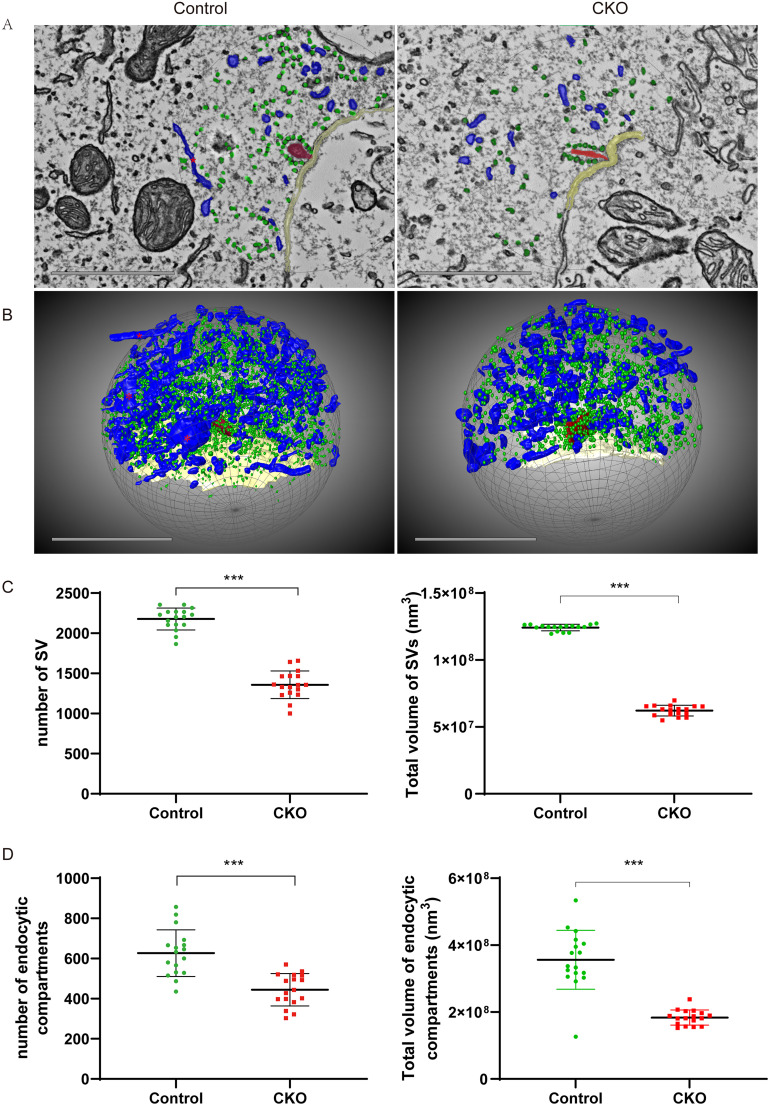
Number count and total volumes of the SVs and endocytic compartments within radius of 1 μm from IHC ribbons of one representative *Dmxl2* CKO mouse and control mouse (17 ribbons each) at 1 month of age by ATUM-SEM. ***A***, Scanning images of the ultrathin sections of the IHCs showing the analyzed space within radius of 1 μm from the ribbon center. Scale bars: 1 μm. ***B***, 3D reconstruction of the SVs (diameter ∼40 nm, shown in green), endocytic compartments (diameter ≥ 70 nm, shown in blue) within the analyzed space. Scale bars: 1 μm. ***C***, The CKO IHCs have significantly less SVs and endocytic compartments. ***D***, The total volumes of SVs and endocytic compartments are both significantly lower in the CKO IHCs. The data are statistically analyzed using unpaired *t* test. The SVs and endocytic compartments within radius of 1 μm from IHC ribbons of the *Dmxl2* CKO (5 mice, 25 ribbons) and control mice (5 mice, 26 ribbons) are also semiquantitatively analyzed by TEM at 1 month of age (Extended Data Fig. 5-1). By ATUM-SEM, we also analyzed the volume of each individual endocytic compartments within radius of 1 μm from one representative IHC ribbon of the *Dmxl2* CKO and control mice at 1 month of age (Extended Data Fig. 5-2).

Immunofluorescence staining and conventional scanning electron microscopy showed that the *Dmxl2* CKO mice have normal hair cell count, ribbon synapse count, and stereocilia morphology (Fig. 3, *n* = 25–34 each, *p *> 0.05). Semiquantitative transmission electron microscopy analysis, however, reveals that the CKO IHCs have less SVs (*p < *0.001) and reduced total volume of endocytic compartments (*p < *0.01) within radius of 1 μm from the ribbons (Extended Data Fig. 5-1, *n* = 25–26 each). To quantitatively examine the defects of synaptic membrane trafficking at the ultramicrostructure level, we applied automated tape-collecting ultramicrotome scanning electron microscopy (ATUM-SEM) for 3D reconstruction of the IHCs in representative CKO and control mice (Figs. 4, 5, Movies 5-3–5-5). In CKO IHCs, a portion of the CKO ribbons (such as the one shown in Fig. 4*A* and marked as the triangle in Fig. 4*B**–E*) have elongated ribbon bodies that are not seen in the control ribbons (Fig. 4*B*, *n* = 17 each, *p < *0.01), with the number of ribbon-associated SVs (RA-SVs; marked as green in Fig. 4*A*) increases proportionally (Fig. 4*E*). On the contrary, within radius of 1 μm from the ribbon (as illustrated in Fig. 5*A* and Extended Data Fig. 5-1*B*), the CKO IHCs have significantly less SVs and endocytic compartments than the control IHCs (Fig. 5*C,D*, *n* = 17 each, *p < *0.001). The total volumes of SVs and total and individual volumes of endocytic compartments are also reduced in the CKO IHCs (Fig. 5*C,D*, *n* = 17 each, *p < *0.001, and Extended Data Fig. 5-2, *n* = 160–180 each, *p < *0.01). These results suggest that DMXL2 deficiency in IHCs leads to reduced level of synaptic membrane trafficking and depleted reserve pool of the SVs.

10.1523/JNEUROSCI.1405-23.2024.f5-1 Figure 5-1 Semi-quantitative TEM analysis of the SVs and endocytic compartments within radius of 1 μm from IHC ribbons of the *Dmxl2* CKO (5 mice, 25 ribbons) and control mice (5 mice, 26 ribbons) at one month of age. (A) Representative TEM images of the IHC ribbon synapses. Asterisks: ribbons, arrowheads: SVs. Scale bars: 200  nm. (B) Representative TEM images of the analyzed area within radius of 1 μm from the IHC ribbons. Scale bars: 1μm, light brown: pre-synaptic and post-synaptic membranes. (C) Number of SVs per analyzed area. (D) Area of endocytic compartments per analyzed area. The data are statistically analyzed using unpaired *t*-test. Download Figure 5-1, TIF file.

10.1523/JNEUROSCI.1405-23.2024.f5-2 Figure 5-2 Volume of each individual endocytic compartments (n = 160∼180) within radius of 1 μm from one representative IHC ribbon of the *Dmxl2* CKO and control mice at one month of age by ATUM-SEM. The data are statistically analyzed using unpaired *t*-test. Download Figure 5-2, TIF file.

### The *Dmxl2* CKO mice have reduced WDR7 and *Otof* expression in IHCs

**Figure 6.**
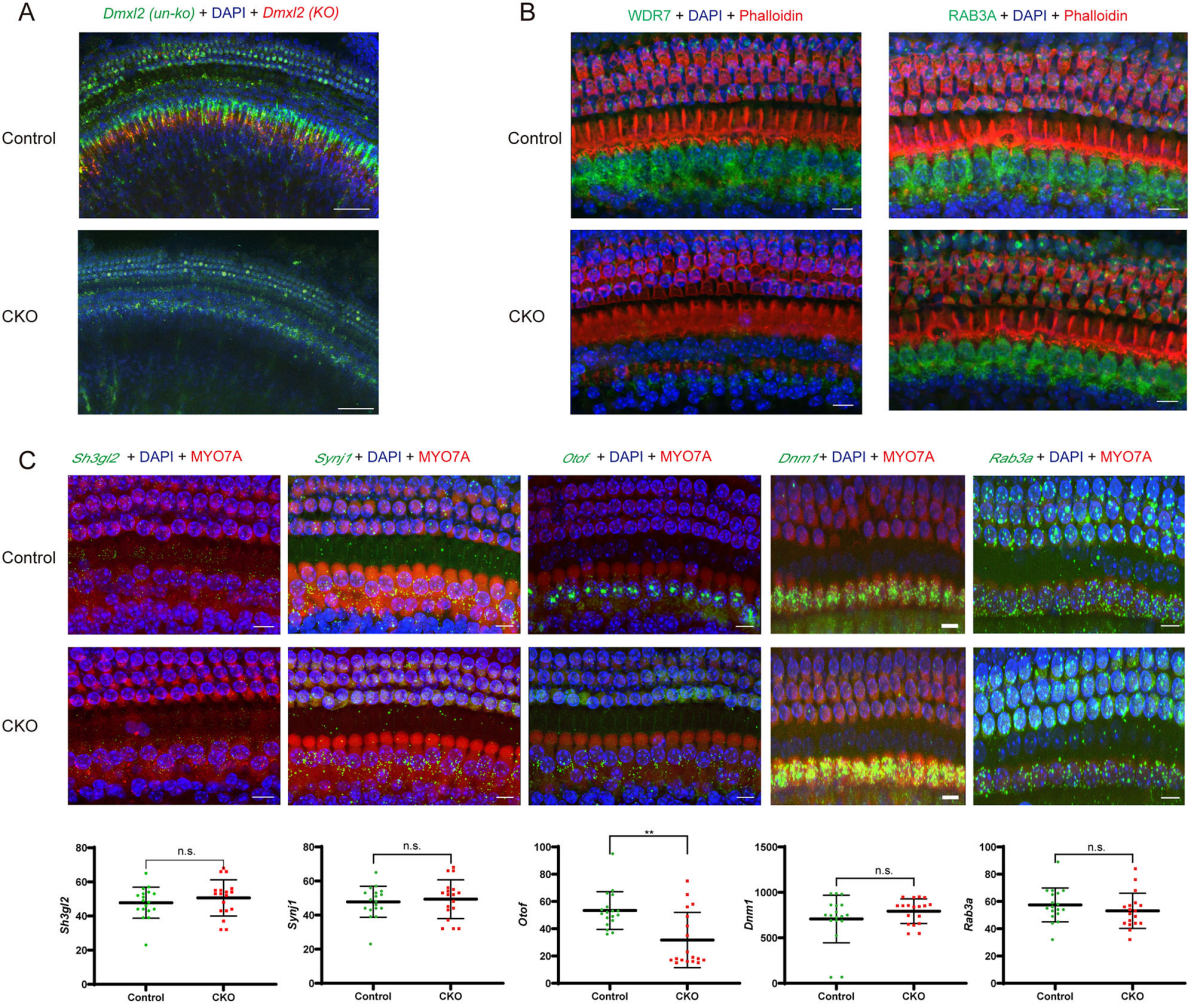
Expression of *Dmxl2*, WDR7 (protein), RAB3A (protein), *Otof*, *Sh3gl2*, *Synj1*, *Dnm1*, and *Rab3a* in the organs of Corti of the *Dmxl2* CKO and control mice at 1 month of age. ***A***, FISH staining shows disrupted *Dmxl2* mRNA expression in the CKO mice. Red and green: probes targeted to (KO) and outside of (un-ko) the deleted exons 2–4 region, respectively. Scale bars: 50 μm. The positions and the sequences of the FISH probes for *Dmxl2* are provided in Extended Data Figure 6-1 and Table 6-2. ***B***, Immunofluorescence staining shows decreased expression of WDR7 and normal expression of RAB3A at the protein level. Scale bars: 10 μm. ***C***, Expression of *Otof* (green) is significantly decreased in the CKO IHCs, while expression of *Sh3gl2*, *Synj1*, *Dnm1*, and *Rab3a* (all in green) remains unchanged at the mRNA level. Please note that the mRNA expression levels are quantified by the number, instead of the fluorescence intensity, of the hybridization dots that represent single mRNA molecules. Red: immunofluorescence staining of MYO7A as the hair cell marker. Scale bars: 10 μm. Examined in three mice each in WT and CKO groups. The data are statistically analyzed using unpaired *t* test.

Loss of *Dmxl2* mRNA expression in hair cells was confirmed by FISH staining of the CKO mouse cochlea (Fig. 6*A*). Immunofluorescence staining of hair cells of the *Dmxl2* CKO mice also showed loss of expression for WDR7 (Fig. 6*B*), the β subunit of rabconnectin 3, and unaltered expression of RAB3A, whose activation is closely regulated by rabconnectin 3 through Rab3 GDP/GTP exchange protein (Rab3 GEP) and Rab3 GTPase-activating protein (Rab3 GAP). We then used RNAscope to further quantitatively analyzed hair cell expression of several other genes with established roles in IHC SV release and resupply. The transcription level of *Otof* (encoding otoferlin) is significantly decreased in IHCs of the *Dmxl2* CKO mice, while that of *Sh3gl2* (endophilin A2), *Synj1* (synaptojanin 1), *Dnm1* (dynamin 1), and *Rab3a* remains unchanged (Fig. 6*C*, *n* = 17 each, *p < *0.01 for *Otof*, *p > *0.05 for the rest).

10.1523/JNEUROSCI.1405-23.2024.f6-1 Figure 6-1 Schematic illustration of the LoxP sites and FISH probes (KO1 and KO2 for the knockout region; un-KO-1 to un-KO-8 for the un-knockout region) for murine *Dmxl2*. Download Figure 6-1, TIF file.

10.1523/JNEUROSCI.1405-23.2024.t6-2 Table 6-2 Probe sequences for the FISH experiment of *Dmxl2*. Download Table 6-2, DOCX file.

**Movie 5-3.** Still and Extended Data Movie 5-3. Illustration of 3D reconstruction of SVs in IHCs based on the ATUM-SEM data. Ribbon, blue; SVs, green. [View online]

**Movie 5-4.** Still and Extended Data Movie 5-4. Representative 3D reconstruction of SVs and endocytic compartments in IHCs of the CKO mice at 1 month of age. [View online]

**Movie 5-5.** Still and Extended Data Movie 5-5. Representative 3D reconstruction of SVs and endocytic compartments in IHCs of the control mice at 1 month of age. [View online]

## Discussion

In this study, characterization of the hair cell CKO mice for *Dmxl2* provided physiological and morphological evidences to support that DMXL2 (rabconnectin 3α) is required for efficient endocytosis and recycling of SVs in IHCs. Like the *Dmxl2* mutant (*stardust*) zebrafish (Einhorn et al., 2012), the CKO mice show only mild hearing loss (Fig. 1), which is consistent with their normal hair cell number, stereocilia morphology, ribbon synapse count, Ca^2+^ influx, and SV exocytosis under short stimulus (Figs. 2, 3). The suprathreshold amplitude of ABR wave I, however, is severely diminished (Fig. 1), a hallmark of auditory synaptopathy often associated with noise-induced and age-related hearing loss (Kujawa and Liberman, 2015; Liberman et al., 2015). It is worth to note that similar hearing phenotype has been reported in the *fitful* mutant mice for dynamin 1, a protein implicated in regulation of IHC endocytosis (Boumil et al., 2010; Neef et al., 2014). Membrane capacitance measurement of the IHCs in the *Dmxl2* CKO mice shows deficient SV resupply, evidenced by reduction of sustained exocytosis under prolonged stimulus (Fig. 2*B,C*). Importantly, slowed endocytic membrane retrieval is confirmed by the IHC patch-clamp recordings (Fig. 2*D*) of the *Dmxl2* CKO mice, which has been reported in only a handful of genetic mouse models with deficiency in endocytic proteins dynamin 1 and endophilin A (Neef et al., 2014; Kroll et al., 2019). Other than IHCs, breeding the *Dmxl2* CKO mouse with the *Gfi1*-cre mouse is anticipated to result in the deletion of *Dmxl2* in OHCs and vestibular hair cells as well. However, by balanced beam tests, rotarod tests, DPOAE, and NLC analysis, the present study does not identify any vestibular or OHC dysfunction for the *Dmxl2* CKO mice (Extended Data Figs. 1-1, 1-2).

To further investigate how compromised endocytic membrane retrieval affects SV trafficking and maintenance of different vesicle pools in the *Dmxl2* CKO IHCs, we quantitatively analyzed all SVs and endocytic compartments using ATUM-SEM, which enables three-dimensional reconstruction of the IHCs with larger scanning range and better resolution than conventional approaches such as TEM or EM tomography. Together with the semiquantitative TEM analysis, our results showed that DMXL2 deficiency does not attenuate abundance of the ribbon-associated SVs (Fig. 4), but leads to ∼40% reduction in total numbers of the SVs within radius of 1 μm from the ribbons in the *Dmxl2* CKO IHCs (Fig. 5, Extended Data Fig. 5-1), suggesting a reduced reserve pool of vesicles. The existence of a large, distant reserve pool of SVs with distinct release kinetics has been validated in IHCs by several studies, which ensures rapid, Ca^2+^-dependent vesicle recruitment to the release site during sustained stimulus (Schnee et al., 2011; Michalski et al., 2017). Notably, deficiency of synaptojanin 1, a polyphosphoinositide phosphatase regulating clathrin-coated pit formation and SV recycling (Cremona et al., 1999; Perera et al., 2006), also results in decreased reserve pool in IHCs of the mutant zebrafish (Trapani et al., 2009).

In addition to the reduced reserve pool of SVs, our data show that the total volume and individual volume of endocytic compartments are also smaller in the *Dmxl2* CKO IHCs (Fig. 5, Extended Data Figs. 5-1, 5-2). Endosomal sorting is an important step for recycling of SV proteins and membranes, which in IHCs enables sustained exocytosis and neurotransmitter release by recruiting bulk endocytosis and SV reformation to balance the massive exocytosis (Neef et al., 2014). Interestingly, accumulation of ELVs has been observed in IHCs of AP2 or endophilin-A deficient mice, suggesting impaired SV reformation following bulk endocytosis (Jung et al., 2015; Kroll et al., 2019). In contrast, the present study observes reduced endocytic compartments in the *Dmxl2* CKO IHCs, which we hypothesize results from impaired endocytic membrane retrieval during earlier steps of bulk endocytosis.

Previous studies have established otoferlin as a multifaceted, essential protein for hair cell exocytosis. In addition to its role as a Ca^2+^ sensor, otoferlin also functions in SV priming/replenishment and exocytosis–endocytosis coupling (Pangrsic et al., 2010; Duncker et al., 2013; Michalski et al., 2017). In the present study, our RNAscope experiments revealed significantly decreased transcription of *Otof* in the IHCs of the *Dmxl2* CKO mice (Fig. 6). Interestingly, disturbance of sustained exocytosis has been observed in several mouse models with reduced otoferlin expression, including those with specific mutations in *Otof* and knockouts for AP-2μ and endophilin-A (Pangrsic et al., 2010; Jung et al., 2015; Strenzke et al., 2016; Kroll et al., 2019). It is tempting to speculate that the reduction of sustained exocytosis in the *Dmxl2* CKO mice (Fig. 3) is a direct result from decreased *Otof* expression in IHCs. However, unlike most of those mutant mouse models, the number of RA-SVs in the *Dmxl2* CKO mice is not decreased (Fig. 5), suggesting a different underlying mechanism.

Based on the previous and current studies, DMXL2 may have dual functions in auditory hair cells. It has been shown to facilitate assembly and proper synaptic localization of the V-ATPase subunits, ensuring acidification of the functional SVs (Einhorn et al., 2012). On the other hand, our results propose an expanded role of DMXL2 controlling the endocytic membrane trafficking and resupplying of the SVs in IHCs. Combining both findings, DMXL2 is likely a key component of the molecular machinery enabling fast and sustainable release of auditory ribbon synapses, though further studies are required to elucidate specific pathways and interacting proteins of DMXL2 in this aspect. Since auditory synaptopathy is a common feature for noise-induced hearing loss and age-related hearing loss, our results may also shed light into the pathogenic mechanism for both at the molecular level.

## Data Availability

This study includes no data deposited in external repositories.
