# Supplementary figures and images for: DMXL2 Is Required for Endocytosis and Recycling of Synaptic Vesicles in Auditory Hair Cells
Source: J Neurosci. 2024 Aug 15;44(38):e1405232024. doi: 10.1523/JNEUROSCI.1405-23.2024 (PMC11411588; doi:10.1523/JNEUROSCI.1405-23.2024)

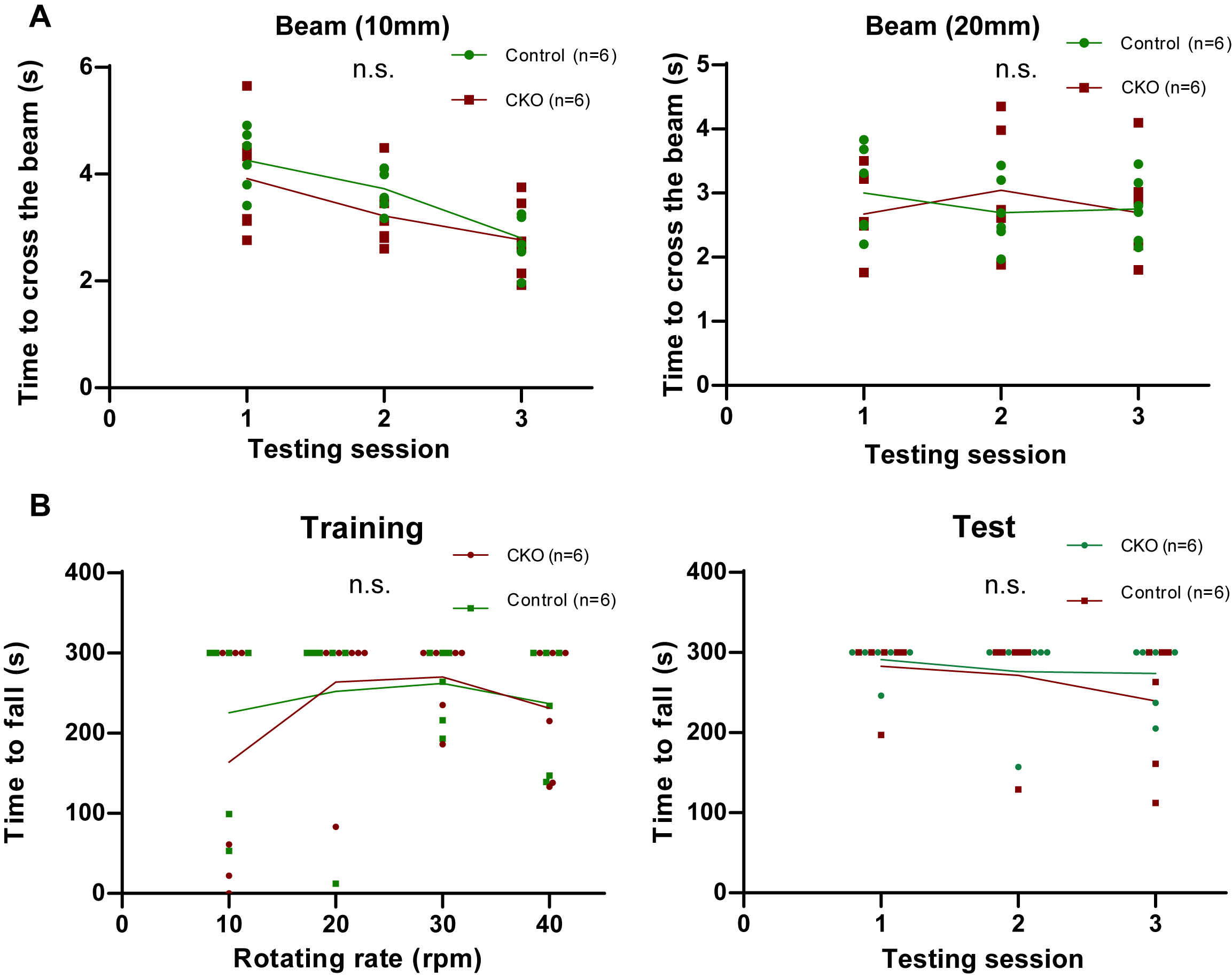

Supplement: Figure 1-1 — Vestibular functions of the Dmxl2 CKO and control mice at one month of age. (A) Time to cross the beam is indistinguishable between the Dmxl2 CKO and control mice in the balanced beam tests. (B) Time to fall is indistinguishable between the Dmxl2 CKO and control mice in the rotarod tests. n: animal numbers. Groups are compared using paired t-test. Download Figure 1-1, TIF file. [file jneuro-44-e1405232024-s001.tif]

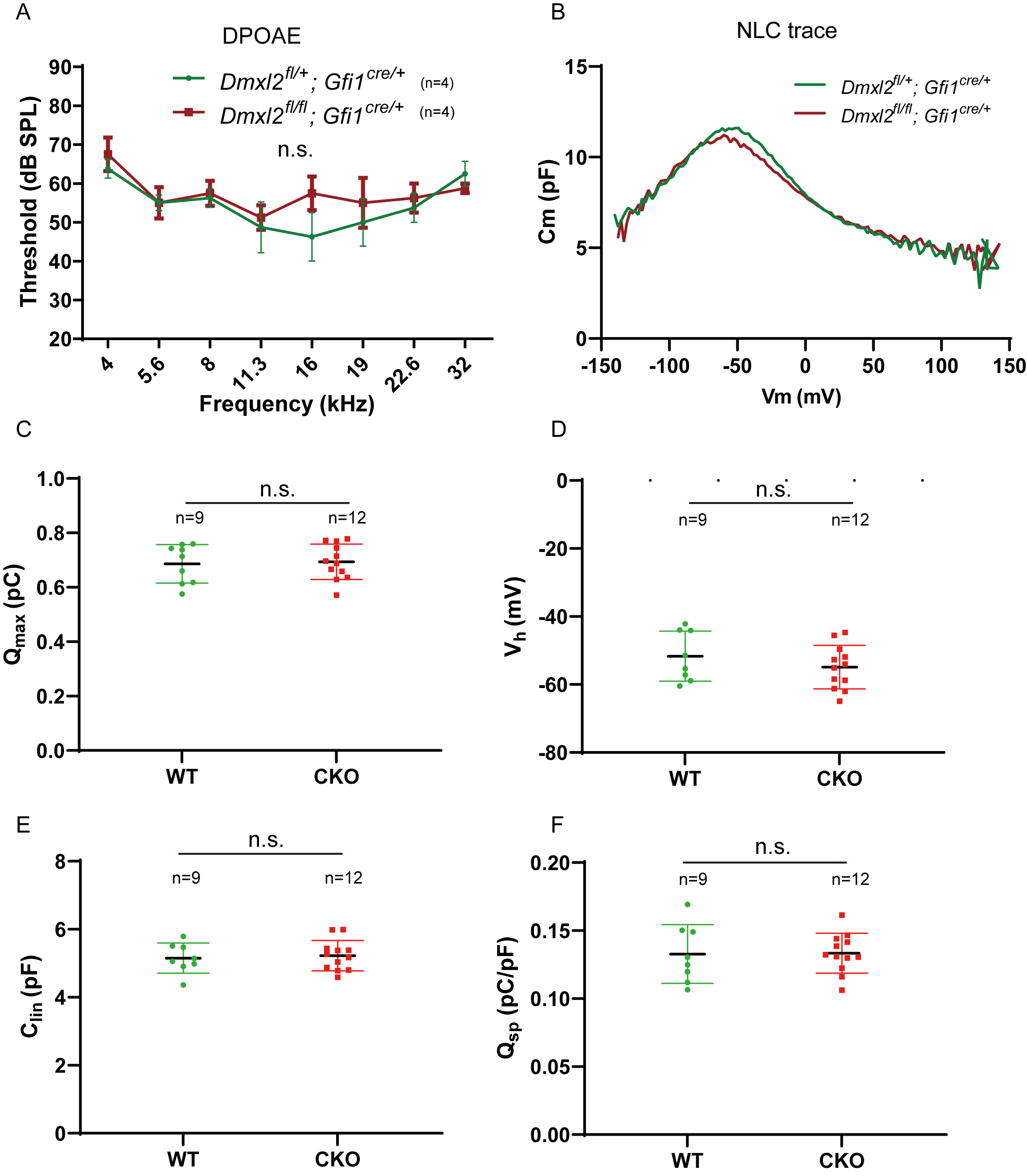

Supplement: Figure 1-2 — OHC functions of the Dmxl2 CKO and control mice at one month of age. (A) The Dmxl2 CKO mice have normal DPOAE thresholds. n: animal numbers. (B-F) Whole cell patch clamped OHCs of the Dmxl2 CKO mice show normal NLC trace, Qmax, Clin, and normalized prestin’s charge density Qsp derived from Qmax/Clin, suggesting normal OHC electromotility. n: OHC numbers; the animal numbers for WT and CKO groups are 5 each. Data are presented as the mean ± SEM and statistically analyzed using unpaired t-test. Download Figure 1-2, TIF file. [file jneuro-44-e1405232024-s002.tif]

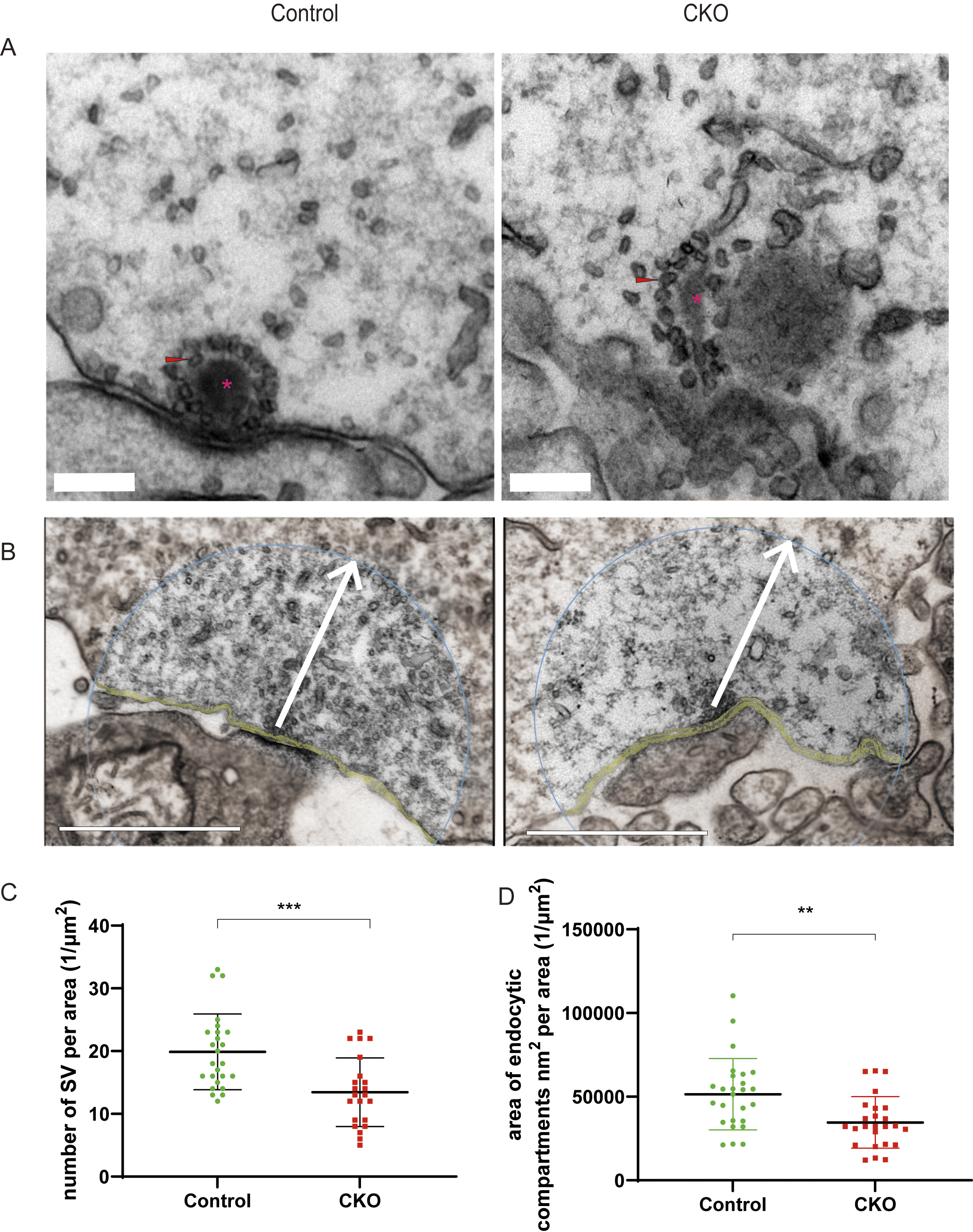

Supplement: Figure 5-1 — Semi-quantitative TEM analysis of the SVs and endocytic compartments within radius of 1 μm from IHC ribbons of the Dmxl2 CKO (5 mice, 25 ribbons) and control mice (5 mice, 26 ribbons) at one month of age. (A) Representative TEM images of the IHC ribbon synapses. Asterisks: ribbons, arrowheads: SVs. Scale bars: 200 nm. (B) Representative TEM images of the analyzed area within radius of 1 μm from the IHC ribbons. Scale bars: 1μm, light brown: pre-synaptic and post-synaptic membranes. (C) Number of SVs per analyzed area. (D) Area of endocytic compartments per analyzed area. The data are statistically analyzed using unpaired t-test. Download Figure 5-1, TIF file. [file jneuro-44-e1405232024-s003.tif]

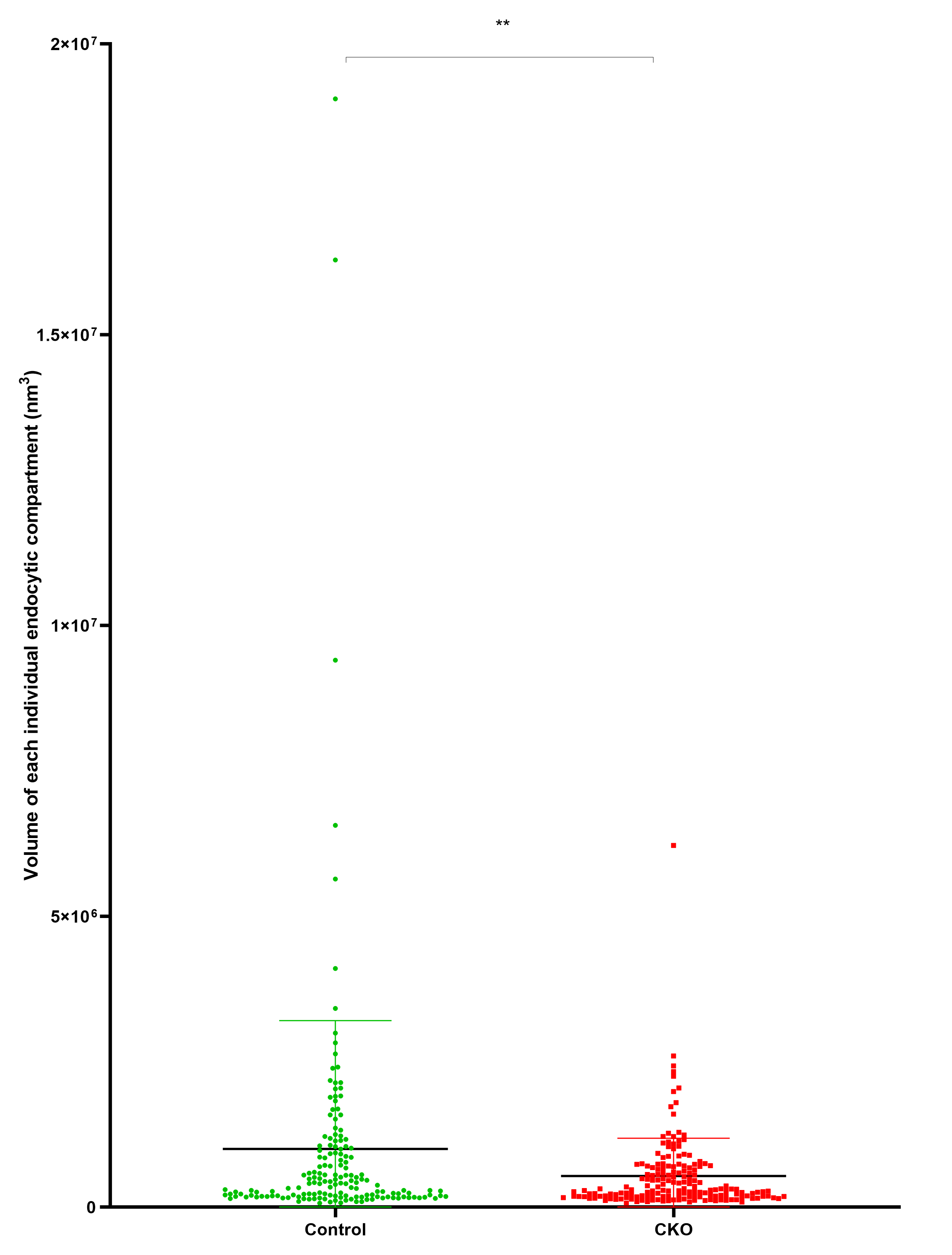

Supplement: Figure 5-2 — Volume of each individual endocytic compartments (n = 160∼180) within radius of 1 μm from one representative IHC ribbon of the Dmxl2 CKO and control mice at one month of age by ATUM-SEM. The data are statistically analyzed using unpaired t-test. Download Figure 5-2, TIF file. [file jneuro-44-e1405232024-s004.tif]

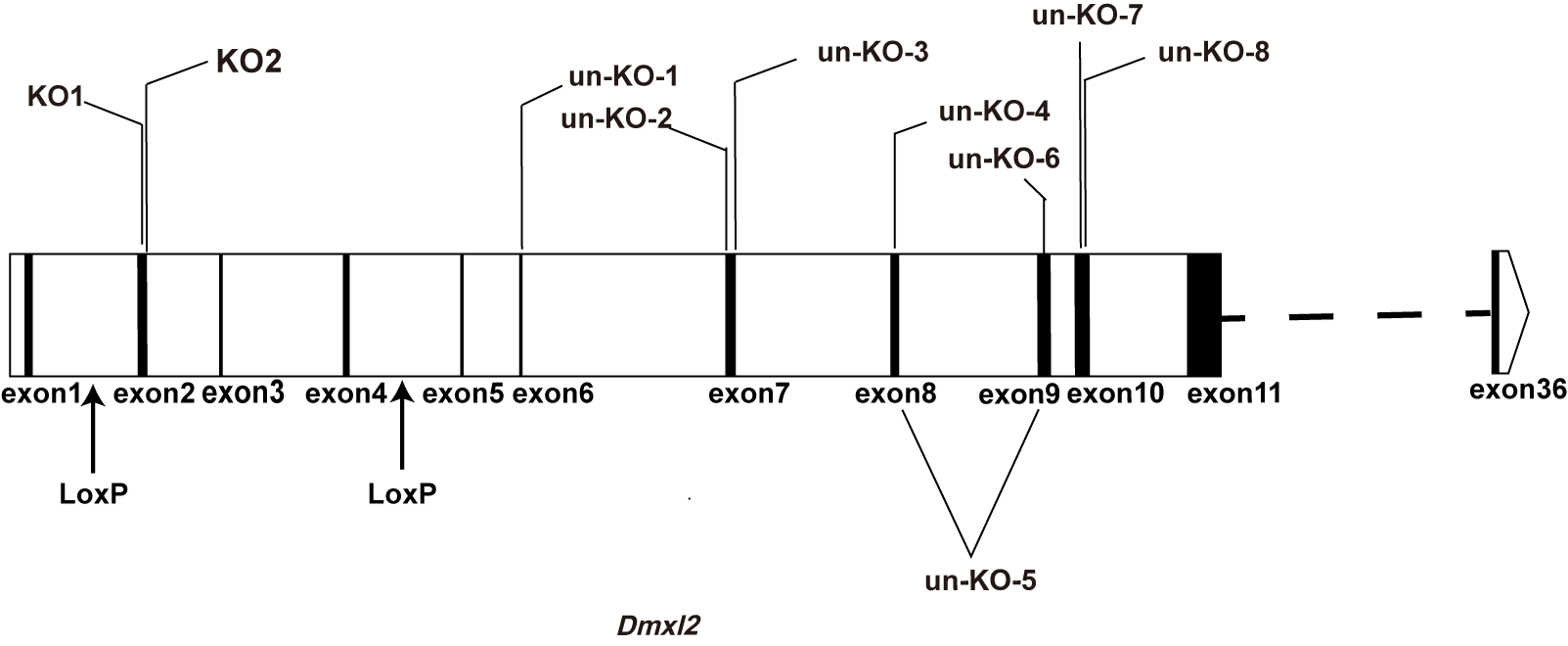

Supplement: Figure 6-1 — Schematic illustration of the LoxP sites and FISH probes (KO1 and KO2 for the knockout region; un-KO-1 to un-KO-8 for the un-knockout region) for murine Dmxl2. Download Figure 6-1, TIF file. [file jneuro-44-e1405232024-s011.tif]
