# Supplementary material for: DMXL2 Is Required for Endocytosis and Recycling of Synaptic Vesicles in Auditory Hair Cells
Source: J Neurosci. 2024 Aug 15;44(38):e1405232024. doi: 10.1523/JNEUROSCI.1405-23.2024 (PMC11411588; doi:10.1523/JNEUROSCI.1405-23.2024)
Supplement: Table 6-2 — Probe sequences for the FISH experiment of Dmxl2. Download Table 6-2, DOCX file. [file jneuro-44-e1405232024-s012.docx]

**Table 6-2.** Probe sequences for the FISH experiment of *Dmxl2*.

| Probe | Sequences |
| --- | --- |
| ko-1 | 5’-AGTCACTCGCCAGAATAACAATATCACA-3’ |
| ko-2 | 5’-CCACACAACTGACTTGGATGTTCC-3’ |
| un-ko-1 | 5’-TCACAAATCTGCTCACCCAAAAGG-3’ |
| un-ko-2 | 5’-CTTAAATTCTTCAAATGGTGTATTGTCTC-3’ |
| un-ko-3 | 5’-TATCTGTAGTAGGATTGATGCTCGCC-3’ |
| un-ko-4 | 5’-CAGAAAGTTTTCGTAATTGGTGCATAA-3’ |
| un-ko-5 | 5’-ATCCAGAGATAATTCATGGTCCAGTTT-3’ |
| un-ko-6 | 5’-CTCTGTCAGCAGCGTTTCAATCTTC-3’ |
| un-ko-7 | 5’-AACAGGAATCCGAGAGGAAAAAGAA-3’ |
| un-ko-8 | 5’-GGGTCTACACGCCACAAAATAAGTT-3’ |
